# Effective Fabrication of Graphene-Coated Ionic Polymer Membrane Actuators

**DOI:** 10.3390/polym17233170

**Published:** 2025-11-28

**Authors:** Kiwon Park

**Affiliations:** Department of Mechanical & Automotive Engineering, Youngsan University, Junam-ro 288, Yangsan-si 48015, Republic of Korea; kwp@ysu.ac.kr

**Keywords:** ionic polymer–metal composite (IPMC), soft actuator, graphene electrode, microwave treatment, electromechanical modeling

## Abstract

Ionic polymer–metal composites (IPMCs) are promising soft actuators; however, they face challenges such as solvent evaporation, low blocking force, and complex fabrication processes. This study introduces a simplified method for fabricating ionic polymer–graphene composite (IPGC) actuators using Nafion 117 membranes and graphene powder. Graphene was directly rubbed onto the membrane surface and subjected to brief microwave irradiation to form durable electrodes, eliminating the need for solvents, multilayer casting, or expensive metal plating. The experimental results indicated that repeated fabrication cycles reduced surface resistance and enhanced bending performance, with optimal displacement achieved after three cycles. Scanning electron microscopy confirmed improved adhesion and surface uniformity following microwave treatment. A hybrid electromechanical model, combining an RC circuit with a mass–spring–damper system, was developed to accurately predict the static behavior of the actuator and achieve reliable parameter estimation. Although the bending performance of the ionic polymer actuator fabricated using the proposed method reaches approximately 75% of that of conventionally produced IPMCs, the method offers a significantly simpler and lower-cost fabrication process.

## 1. Introduction

**Figure 1:**
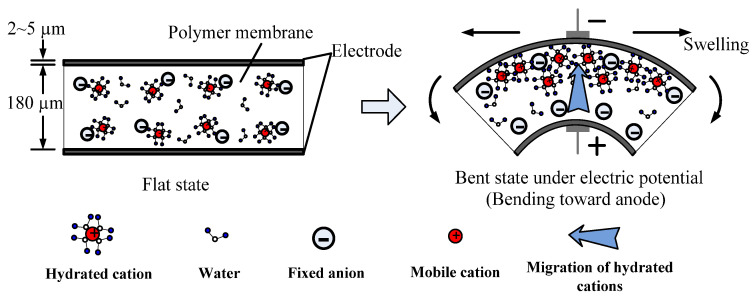
Bending mechanism of IPMC actuator.

Ionic polymer–metal composites (IPMCs) have attracted significant attention as soft actuators owing to their unique ability to undergo substantial bending deformation under low applied voltages [1,2,3]. They are a class of electroactive polymer actuators comprising an ion-conducting polymer membrane, typically Nafion, sandwiched between conductive electrodes. The bending behavior of IPMC actuators is primarily attributed to the migration of cations and water molecules within the polymer matrix when subjected to an electric field, which leads to asymmetric swelling and subsequent bending toward the anode (Figure 1). This characteristic renders them highly suitable for applications in soft robotics, biomedical devices, and adaptive structures [1,2,3].

Despite their potential, several persistent challenges hinder practical deployment. One major issue is solvent evaporation from the polymer matrix, which degrades the bending performance and causes instability over time. This is critical because IPMCs rely on hydrated ionic channels for actuation, and water loss significantly reduces ionic conductivity and actuation efficiency [4]. Another limitation is their inherently low blocking force, which typically reaches only a few milliNewtons—far below the levels required for many practical applications [5]. In addition, conventional fabrication methods for IPMCs involve multiple complex steps, including solution casting, hot pressing, and time-consuming electroless plating. These procedures are labor-intensive and expensive, posing scalability issues for mass production [6,7].

To address the limitations of conventional IPMCs, studies have investigated the incorporation of carbon nanomaterials, such as carbon nanotubes (CNTs) and doped graphene oxide, into either the polymer matrix or electrode layers to enhance the conductivity, mechanical strength, ion retention, and blocking force. For example, multiwalled carbon nanotubes (MWCNTs) mixed with a Nafion solution have been cast in Teflon molds to create ion-conductive films exhibiting greater deflection at high frequencies [8]. CNTs and carbon black films have also been hot-pressed onto Nafion membranes to improve the blocking force and bending displacement [7]. Zhang et al. reported that adding sulfonated graphene oxide (SGO) significantly increased the tip displacement and blocking force in relation to those observed in pure Nafion-based IPMCs [8].

Graphene has also been used to fabricate electrodes. Techniques include spraying solvents of MWCNT–graphene mixtures onto Nafion membranes followed by baking [9] and applying reduced graphene oxide or directly grown graphene via electrospray coating and wet-transfer methods [10]. Alternative approaches involve dip-coating Nafion membranes with polyaniline–graphene mixtures to avoid expensive, time-consuming Pt plating [11] and coating graphene–Nafion blends onto membranes to form electrodes [12]. However, most of these methods involve significant fabrication complexities, e.g., preparing graphene-based solvent mixtures, casting multilayer structures, and functionalizing nanomaterials.

The objective of this study was to develop a significantly simpler yet effective fabrication method for IPMCs, which is intended to replace the conventional complex and laborious processes that remain a major obstacle to the further advancement of IPMC-based technologies. I propose a novel process that uses a commercially available Nafion 117 film and graphene powder for streamlined fabrication while maintaining performance. In this approach, graphene powder is directly applied to a dry Nafion surface and gently rubbed with a gloved finger for uniform coverage. The film is then briefly exposed to microwave radiation to enhance the graphene adhesion and form more durable surface electrodes. This process eliminates complex solvent preparation, multilayer casting, and nanomaterial functionalization, significantly reducing the fabrication time and enabling scalability. Furthermore, a hybrid model comprising an electrical circuit and a mechanical system was developed in a cantilevered configuration to evaluate the actuator performance. The model accurately predicted the tip deflection of the IPGC, confirming the feasibility of the proposed fabrication method.

The technology presented herein can significantly simplify the complex conventional fabrication process of IPMCs. Furthermore, by replacing expensive surface electrode materials with inexpensive carbon-based materials, the proposed method can reduce the production cost of ionic-polymer-based actuators. Therefore, the contribution of this study lies in presenting a more accessible fabrication approach that can promote the wider adoption and practical utilization of ionic polymer actuators.

## 2. Fabrication of Graphene-Coated Ionic Polymer Actuator

With the discovery of the actuator property of IPMCs in the early 1990s, diverse fabrication approaches for IPMCs, including methods using Au and Ag particles as surface electrode materials and techniques to develop patterned surface electrodes, have been proposed [13,14,15]. Although these methods improve the actuation properties of IPMCs, the requirement for advanced fabrication techniques limits the application of IPMC actuators in various research areas. This study presents a simple fabrication method involving the substitution of metallic surface electrode materials with graphene.

The conventional fabrication method using Pt as the surface electrode material involves plating the surface electrode electrochemically in two steps [3,16]. The Nafion membrane is a hydrophilic porous material comprising backbone polymers terminating in negatively charged sulfonic groups (SO_3_^−^); hence, it only absorbs positive ions. In the first step, the Nafion membrane is immersed in a chemical solution, and Pt ions (Pt^+^) combine with the sulfonic groups of the backbone polymers on the surface of the Nafion membrane through chemical reactions. The depth of the Pt^+^ permeation layer increases according to the number of repetitions of the first step, and the typical permeation depth is approximately <10 µm. In the second step, Pt ions (Pt^+^) are plated onto the outer surface of the Nafion membrane without permeating the surface layer. This reduces the surface resistance of the electrode, facilitating transmission of the voltage applied on the surface layer to the inner layer of the IPMC. In the conventional two-step fabrication method, the Nafion membrane is fully hydrated and expanded in a chemical solution; therefore, the IPMC is fabricated in its original flat shape.

After fabrication, the IPMC is immersed in a variety of ionic liquids—typically saline water—for water and Na^+^ ions to be absorbed by the membrane. The second step of the fabrication procedure facilitates the transmission of the electric potential applied to both surface electrodes of the IPMC to the inner layer of the IPMC. This induces positive-ion movement toward the cathode, which causes asymmetric swelling of the cathode-side membrane in relation to the anode-side membrane, leading to bending motion in IPMC actuators. Hence, effective fabrication of the IPMC should satisfy the effects of the two-step fabrication method.

Flexible actuators were fabricated using a Nafion 117 membrane and graphene powder. There are several reasons for selecting powder-type conductive materials. First, Nafion is a hydrophilic material; hence, it is distorted even when contacted by human sweat. The use of a powder-type material can prevent physical distortion of the Nafion membrane during fabrication. Second, graphene has high electrical conductivity of up to 6000 S cm^−1^ [17,18]. Third, graphene is a thin-layered material that is held together well by strong covalent bonds, which can increase the durability of the surface electrode [19].

**Figure 2:**
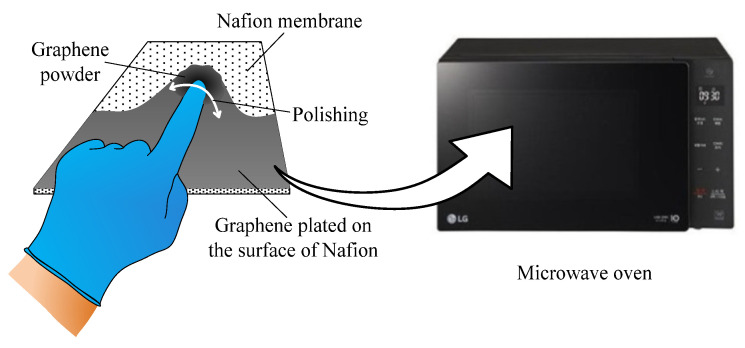
Fabrication procedure for the graphene-powder-coated IPGC.

Table 1 presents the properties of the graphene powder supplied by the manufacturer. The advantage of the proposed fabrication method is its simplicity, as complex chemical reactions are not required. The ionic polymer–graphene composite (IPGC) actuator was fabricated by manually applying graphene powder onto both sides of Nafion membrane using a gloved finger. The membrane was then placed in a microwave oven and exposed to 200-W microwaves at a frequency of 2.45 GHz on both sides for 4 s each. Figure 2 illustrates each step of the fabrication procedure.

Previous research has demonstrated that microwave radiation can effectively induce carbon-based coatings on polymer surfaces [20], motivating the use of similar techniques to enhance electrode adhesion in IPGC.

Microwave irradiation can rapidly heat graphene layers due to their strong microwave absorption capability, leading to localized high temperatures that induce surface oxidation, defect generation, and partial particle rearrangement [20,21]. Depending on the ambient environment, either reduction or oxidation of graphene may occur. In air or oxygen-rich atmospheres, the high local temperatures promote reactions between graphene and atmospheric oxygen, resulting in the formation of oxygen-containing functional groups such as hydroxyl, epoxy, and carboxyl groups on the graphene surface [21]. These oxygenated groups enhance surface polarity and improve interfacial adhesion through hydrogen bonding or electrostatic interactions with the ionic sulfonic acid (SO_3_^−^) groups of the Nafion membrane [22]. In addition, microwave-induced particle rearrangement and mild oxidation at defect sites can increase the effective contact area and mechanical interlocking between the carbon layer and polymer surface [23].

Conversely, when graphene is exposed to microwaves in an inert or vacuum atmosphere, rapid thermal decomposition of preexisting oxygen groups occurs, leading to reduction of graphene oxide (GO) to reduced graphene oxide (rGO) and restoration of sp^2^-hybridized carbon networks [24,25]. This dual nature of the microwave–graphene interaction allows selective tuning of surface functionality depending on the processing environment. In the present fabrication approach, mild surface oxidation induced by microwave treatment improves the interfacial bonding of graphene to the polymer surface, forming a conductive interface that serves as the electrode layer of the IPGC.

In this study, a fabrication cycle involving the application of graphene powder to both sides of a Nafion membrane, followed by microwave irradiation, was performed multiple times to reduce the surface resistance of the electrode layers. This approach provides a simplified alternative to the second step of conventional fabrication processes.

The fabricated IPGC, produced through the described fabrication procedure, was immersed in saline water with an NaCl concentration of 0.9% to allow for sufficient absorption of Na ions before being used for the experiments.

## 3. Experimental Setup

**Figure 3:**
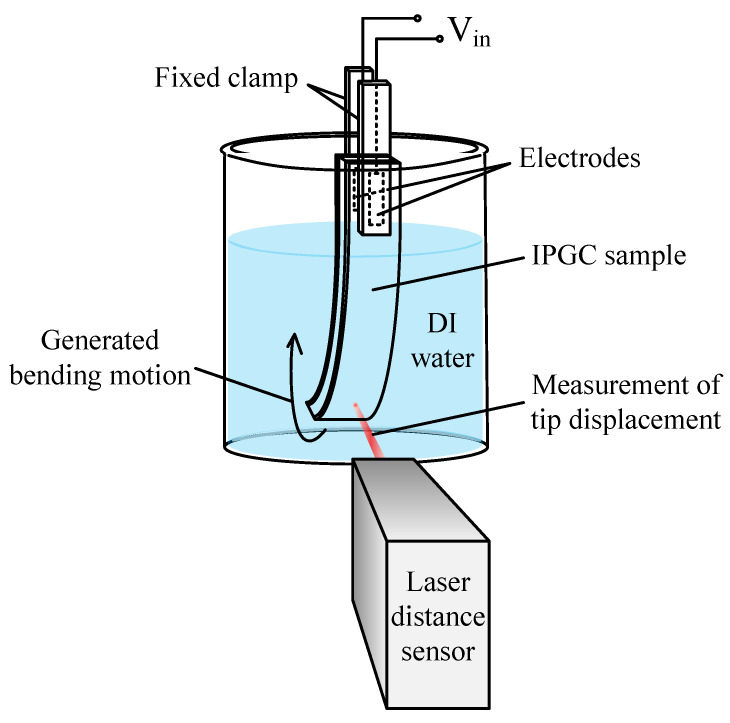
Experimental environment.

An experimental setup (Figure 3) was constructed to evaluate the performance of the fabricated IPGC. The IPGC sample was immersed in deionized (DI) water and fixed using a clamp. The electrodes were attached to the contact surfaces of the IPGC and connected to a power source. A laser sensor was used to measure the tip displacement. During the bending measurement, the sample was immersed in DI water to prevent evaporation of the internal solvent, which is critical for maintaining the lifespan of the IPGC. The signals transmitted by the laser sensor were collected using the NI-DAQ system at a sampling frequency of 1 kHz and analyzed using the MATLAB^®^ DAQ Toolbox (Mathworks^®^, Natick, MA, USA).

## 4. Modeling of IPGC

Early modeling studies investigated the transport of hydrated cations and water molecules in the Nafion membrane under an applied electric field [26,27]. These approaches, which are typically based on the Nernst–Planck equation or coupled ion–water transport formulations, describe the charge redistribution, osmotic pressure gradients, and electroosmotic drag that induce bending deformation. However, although charge-based models provide detailed physical insight, they are mathematically complex and require numerical solutions. Hence, equivalent circuit models have been proposed to provide simplified yet practical representations that approximate ion migration and charge accumulation using lumped electrical elements [3,28]. Resistive and capacitive components are commonly employed to represent ionic conduction in the bulk membrane and charge storage at the electrode–polymer interface, respectively. These circuit models are computationally efficient and can be integrated with mechanical analogs, such as mass–spring–damper systems, to construct hybrid electromechanical models. Such models have proven effective in predicting actuator dynamics, including tip displacement and relaxation behavior.

**Figure 4:**
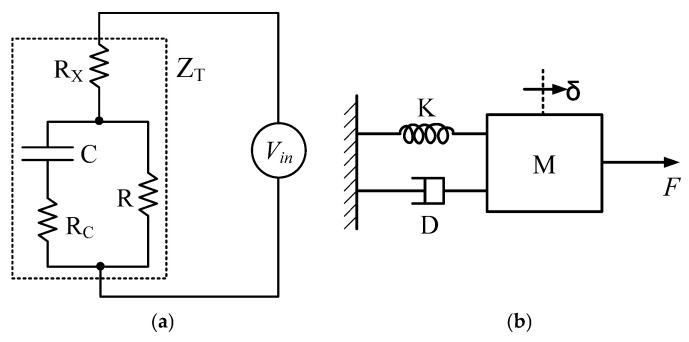
Diagrams for modeling the IPGC actuator: (**a**) circuit diagram for analyzing inner charge movement and (**b**) force diagram for analyzing the relationship between the generated force and tip deflection.

Figure 4a illustrates the circuit model employed to estimate the variation in the motion of internal cations when a voltage is applied to the surface electrodes of an IPGC fixed in a cantilever configuration. As described previously, the application of a potential difference across the IPGC surface induces the migration of cations bound to water molecules inside the polymer matrix. Consequently, the imbalance in the charge distribution between the internal regions is analogous to the charge imbalance across a capacitor during charging.

Furthermore, the electrostatic equilibrium established inside the Nafion film, arising from the attractive forces between anions (SO_3_^−^) in the polymer backbone and cations, as well as the repulsive interactions among like-charged ions, can be represented by the discharge behavior of an RC circuit comprising resistors connected in series and parallel with a capacitor. Therefore, the migration of cations inside the IPGC under electrical stimulation can be modeled using a circuit comprising electrical elements that reflect the structural characteristics of the material.

The equivalent circuit model of the IPGC consisted of composite impedance elements arranged in two parallel branches across the input voltage. In this RC network, the capacitor C represents the charging phenomenon due to the charge imbalance between the Nafion film and its surfaces. The resistor R_C_ describes the ionic migration resistance, whereas R represents the intrinsic resistance of the Nafion film. The RC network was connected in series with the film resistance R_X_ relative to the voltage source. By applying the Laplace transformation, the total equivalent impedance (Z_T_) of the R–C elements can be obtained:

(1) $$Z_{T}=\left(\frac{1}{Cs}+\;R_{C}\right)∥R\;+\;R_{X}.$$

Accordingly, the current flowing inside the IPGC is expressed as

(2) $$I_{i}\left(s\right)=\frac{V_{i}(s)}{Z_{T}}.$$

The motion of the internal cations is then obtained by differentiating Equation (2), resulting in Equation (3).

(3) $$Q_{i}\left(s\right)=\frac{\left(R+R_{C}\right)Cs+1}{\left(RR_{C}+R_{X}R_{C}+RR_{X}\right)Cs^{2}+\left(R+R_{X}\right)s}\;V_{i}\left(s\right)$$

Next, to describe the relationship between the bending moment induced by cationic motion inside the IPGC and the resulting tip deflection, a mechanical second-order system is considered (Figure 4b). In this model, *F* denotes the bending moment generated at the IPGC tip owing to cation migration, and *δ* represents the tip deflection. M represents the effective mass of the IPGC, and K and D denote the spring constant inducing vibration and the damping coefficient attenuating vibration, respectively. The relationship between the applied force at the IPGC tip and the resulting deflection is expressed as

(4) $$F\left(s\right)=Ms^{2}\delta \left(s\right)+Ds\delta \left(s\right)+K\delta \left(s\right)I_{i}\left(s\right)=\frac{V_{i}\left(s\right)}{Z_{T}}.$$

Furthermore, assuming that the force *F* generated at the tip is proportional to the velocity of cation migration, the relationship between the migration velocity and the induced force is given by Equation (5), where α is a proportional constant.

(5) $$F\left(s\right)=\alpha sQ_{i}(s)$$

By applying Equations (3) and (4) to Equation (5), the following transfer function between the input voltage (*V_i_*(*s*)) and the tip deflection (δ(s)) of the IPGC is obtained.

(6) $$\frac{\delta (s)}{V_{i}(s)}=\alpha \left(\frac{\left(R_{C}+R\right)Cs^{2}+s}{\left(RR_{C}+R_{X}R_{C}+RR_{X}\right)Cs^{2}+\left(R+R_{X}\right)s}\right)\left(\frac{1}{Ms^{2}+Ds+K}\right)$$

In this study, the MATLAB Parameter Estimation Toolbox was employed to estimate the parameters of the proposed circuit model. Instead of directly estimating the values of M, D, and K, the roots (β and γ) of a quadratic polynomial constructed from these coefficients were estimated to reduce the computational effort.

## 5. Results and Discussion

**Figure 5:**
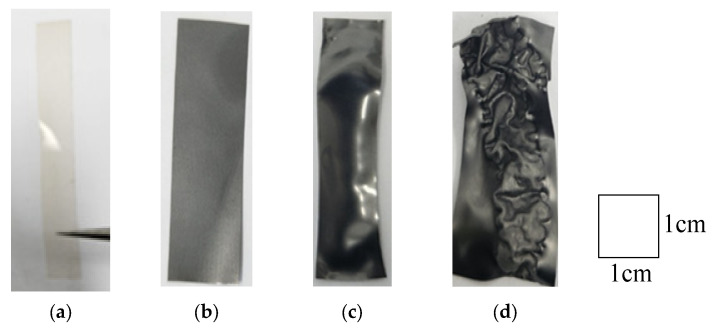
Images of IPGC samples: (**a**) Nafion membrane; (**b**) IPGC sample coated with graphene powder via polishing, as shown in Figure 2; (**c**) IPGC sample coated with graphene powder and exposed to microwave irradiation for 5 s; (**d**) IPGC sample coated with graphene powder and exposed to microwave irradiation for 6 s.

Figure 5 presents images of the graphene-coated Nafion membrane before and after microwave exposure. As shown in Figure 5b,c, the surface appeared brighter and slightly shinier after microwave treatment compared to the pre-exposure state.

**Figure 6:**
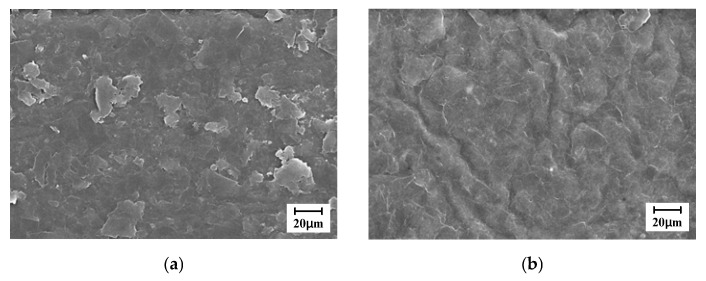
Surface SEM images of IPGC samples (**a**) without microwave exposure and (**b**) with microwave exposure for 5 s.

Scanning electron microscopy (SEM) was employed to closely examine the surface morphology of the graphene-coated membranes before and after microwave exposure. The sample was sputter-coated with Pt, and SEM images were acquired using a SU8220 cold field-emission scanning electron microscope at an accelerating voltage of 5 kV. In Figure 6, the surface exposed to microwave irradiation appears to be significantly smoother than that of the untreated sample, with all the graphene particles firmly attached to the membrane.

**Figure 7:**
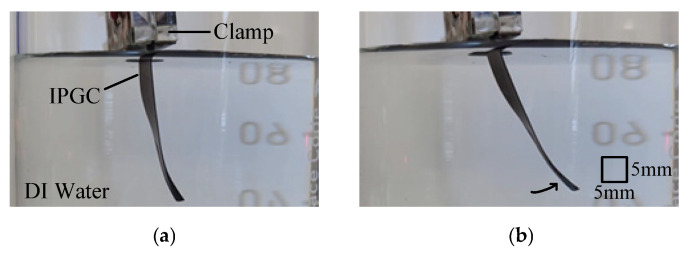
Bending performance (**a**) before and (**b**) after the application of 5 V.

The bending performance of the fabricated IPGC samples was evaluated by measuring the tip displacement under an applied voltage of 5 V across the surface electrodes. Figure 7 shows the variation in the tip deflection of the sample before and after the 5-V input.

**Figure 8:**
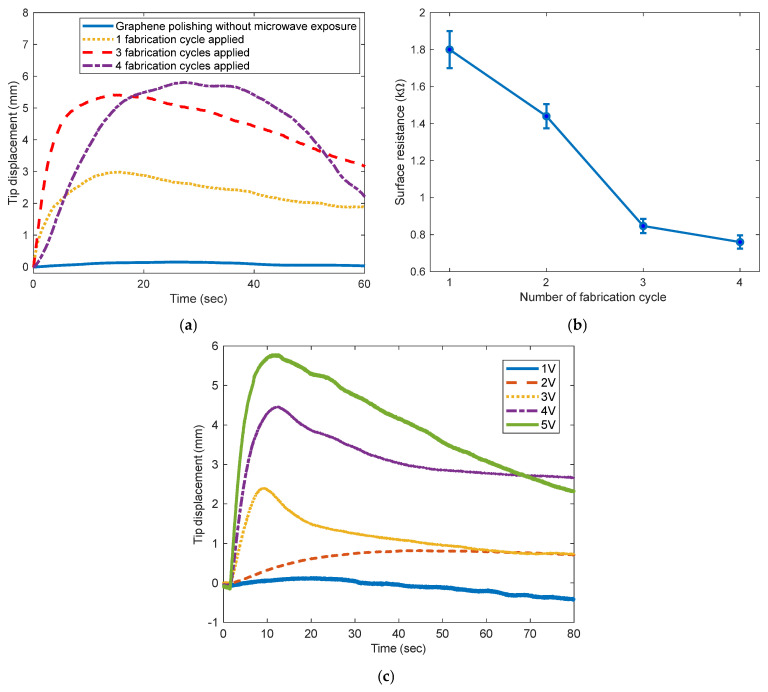
Comparison of tip deflection and surface resistance variations of IPGC samples fabricated with different numbers of fabrication cycles and tip displacement variations per volt: (**a**) tip deflection variations, (**b**) surface resistance variations, and (**c**) tip displacement variations.

The fabrication cycle, consisting of graphene-powder polishing followed by microwave irradiation, was repeated up to four times to reduce the surface resistance. Figure 8a shows the variations in the tip displacement of the IPGC samples. All samples exhibited a back-relaxation phenomenon after reaching their maximum bending displacement. Furthermore, the magnitude of the tip displacement increased with the number of fabrication cycles.

Additional application of carbon-based materials and repeated microwave exposure may induce particle rearrangement within the coating, resulting in the densification of the electrode layers and enhanced bending performance of the ionic polymer composite.

However, after three fabrication cycles, the increase in maximum tip displacement became notably limited. When graphene powder is applied to both the surfaces and exposed to microwave irradiation more than four times, the coating layers may become excessively thick, reducing the flexibility of the electrodes. Consequently, in relation to those of the samples subjected to three additional cycles, the response time of the IPGC increased, along with the degree of back-relaxation.

Figure 8b presents the variation in the surface resistance of the IPGC samples as the fabrication cycle is repeated one to four times. The surface resistance decreased with an increasing number of fabrication cycles. However, beyond the third fabrication cycle, the reduction in surface resistance became less pronounced. This trend in the surface resistance is consistent with the variation in the tip deflection of the IPGC samples (Figure 8a). Therefore, it can be inferred that the change in surface resistance correlates with the bending performance of the IPGC.

As previously described, when graphene is heated by microwave irradiation in air, oxygen-containing functional groups such as hydroxyl, epoxy, and carboxyl groups are generated on its surface. These oxygenated groups increase the surface polarity and enhance interfacial adhesion through hydrogen bonding or electrostatic interactions with the ionic sulfonic acid (SO_3_^−^) groups of the Nafion membrane [22,29]. In addition, while the interlayer interactions of pristine graphene are governed only by π–π stacking and van der Waals forces, oxidized graphene exhibits stronger interlayer bonding due to polar interactions and hydrogen bonding [30]. The strengthening of interlayer interactions induced by graphene oxidation is reflected in the reduction in surface resistance with increasing number of fabrication cycles, as shown in Figure 8b. In summary, repeated microwave irradiation not only improves the bending performance of the IPGC by enhancing the adhesion between graphene and the ionic sulfonic acid (SO_3_^−^) groups of the Nafion membrane (Figure 8a), but also enhances interlayer bonding among surface graphene molecules, as evidenced by the experimental results (Figure 8b).

Figure 8c shows the variation in tip displacement of the IPGC when different input voltages, ranging from 1 to 5 V in 1-V increments, are applied. In the case of IPMCs, the tip displacement is influenced by various factors, such as the input voltage, thickness and material of the polymer film, moisture content, and thickness and material of the surface electrodes. For the IPGC fabricated in this study, the tip displacement performance reaches approximately 75% of that reported for typical IPMCs. However, considering that its fabrication time is shorter and its fabrication process is far simpler than conventional methods, the developed IPGC has unique practical advantages.

**Figure 9:**
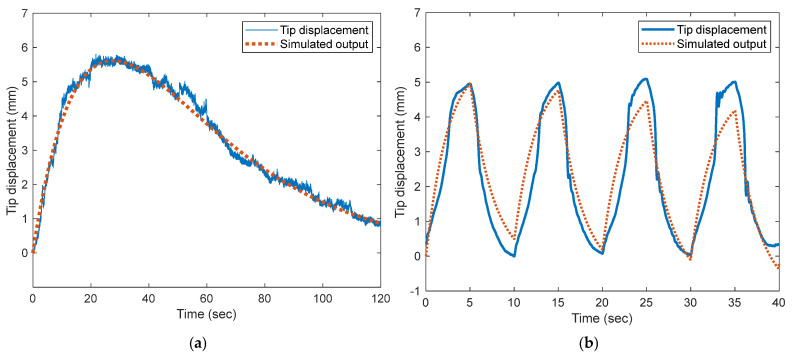
IPGC model performance evaluation: (**a**) tip-deflection estimation under static input and (**b**) tip-deflection estimation under dynamic input.

Figure 9 presents the performance of the IPGC model employed in this study. Table 2 presents the parameter values estimated using the MATLAB Parameter Estimation Toolbox to simulate the output waveforms of the IPGC model.

Figure 9a shows the variation in tip displacement and the corresponding simulated output of the model under a static input of 5 V. Figure 9b shows the dynamic variation in tip displacement and the simulated output of the model when a 5 Vpp, 0.1-Hz square waveform is applied as the input. In Figure 9a, the proposed IPGC model demonstrates excellent performance in representing the static characteristics of the IPGC. However, its accuracy in representing the dynamic characteristics of the IPGC decreased to some extent (Figure 9b).

The simulation results of the IPGC model shown in Figure 9 demonstrate adequate performance in relation to previously reported modeling results. Compared with a similar circuit-based model [31], the proposed model exhibits superior capability in describing the static characteristics. Furthermore, compared to models incorporating charge dynamics [32], the proposed model exhibits better overall performance. Although relatively large errors are observed in representing the dynamic behavior, the performance of the proposed model can be considered appropriate compared to that of similar models reported in the literature [33]. In contrast to previous models, which often exhibit significant errors in describing the bending behavior of IPMCs—particularly in the peak region or during the back-relaxation phenomenon—the proposed model accurately represents the tip displacement of the IPGC over the entire response.

The proposed ionic polymer actuator, which exhibits approximately 75% of the bending performance of conventional IPMCs while offering a remarkably simplified fabrication process, can be applied to a wide range of low-load and cost-sensitive systems. Its potential applications include soft robotic grippers, wearable and biomedical devices, microfluidic pumps and valves, and small-scale positioning mechanisms. Owing to its lightweight structure, flexibility, and ease of mass production, the actuator is particularly suitable for use in soft robotics, portable electronics, and educational or disposable devices where moderate actuation performance is sufficient.

To enable scalable manufacturing, the fabrication process of the proposed ionic polymer actuator can be automated by substituting the manual rubbing and microwave irradiation steps with continuous mechanized operations. The graphene powder can be uniformly deposited on the Nafion surface using a soft nip roller or calendering system that reproduces the mechanical rubbing effect with controlled pressure and speed. Subsequently, the microwave irradiation step can be implemented through a conveyor-type microwave chamber, allowing for precise control of the power density and exposure time to ensure consistent bonding quality. Incorporating in-line monitoring systems such as surface resistance measurement and optical inspection would further enhance reproducibility and process reliability. This automated approach provides a practical route for large-scale, low-cost production of ionic polymer actuators with stable and uniform performance characteristics.

## 6. Conclusions

This paper presents a simplified fabrication method for IPGC actuators that eliminates the complexity of conventional electroless plating and nanomaterial functionalization. By directly applying graphene powder onto Nafion 117 membranes, followed by brief microwave irradiation, durable surface electrodes were formed without the need for solvents or multilayer casting. Surface analyses confirmed that the microwave treatment enhanced the adhesion and uniformity of the graphene layers, resulting in smoother electrode surfaces. The experimental results indicated that repeated fabrication cycles reduced the surface resistance and improved the bending performance, with optimal actuation observed after approximately three cycles. Beyond this point, excessive electrode thickness limits the flexibility and increases back-relaxation effects.

The bending performance of the fabricated IPGC was evaluated in a hydrated state by immersing the sample in DI water, as the actuation characteristics of ionic-polymer-based actuators are highly dependent on the internal water content. Therefore, the performance data presented herein correspond to a fully hydrated condition, and additional degradation mechanisms that may occur upon ambient air exposure have not yet been experimentally investigated. Nevertheless, the simplified dry-type fabrication process of the IPGC, which does not involve electroless plating or metallic deposition, is expected to improve environmental stability against humidity and temperature variations by eliminating residual chemical agents in the electrode layer. Moreover, the microwave irradiation treatment is believed to have enhanced the interfacial adhesion between the graphene powder and Nafion membrane, which may mitigate delamination or surface degradation under cyclic or thermal stress.

Furthermore, a hybrid electromechanical model comprising an equivalent RC circuit and a mass–spring–damper system was developed to predict the tip deflection of the fabricated IPGCs. The model exhibited close agreement with experimental data under static inputs and reasonable accuracy under dynamic excitation, validating its effectiveness for performance evaluation and parameter estimation.

The proposed approach offers significant advantages in terms of fabrication simplicity, scalability, and material cost-effectiveness while maintaining actuation performance comparable to that of conventionally fabricated IPMCs. These findings suggest that graphene-based electrodes combined with microwave-assisted adhesion are promising for practical and high-performance soft actuators in robotics, biomedical devices, and adaptive structures. Future research will focus on optimizing the electrode morphology, extending the solvent retention strategies, and enhancing the long-term stability of IPGC actuators upon continuous operation to further evaluate its applicability in practical environments.

## Figures and Tables

**Table 1:** Properties of the graphene powder.

Manufacturer	cnt@carbonnano.co.kr
Model	GNP-UC
Structure	Multilayer graphene
Number of layers	3–10
Length	5–10 µm
Thickness	3–6 nm
Surface area	150 m^2^/g
Carbon purity	>99%
Production method	Chemical exfoliation (proprietary method)

**Table 2:** Estimated parameter values.

Parameter	α	C (F)	R (Ω)	R_c_ (Ω)	R_x_ (kΩ)	β	γ
Value	15.120	9.852	32.112	788.072	1.016	0.035	0.037

## Data Availability

The data is contained within the article.
